# Occipital Horn Syndrome as a Result of Splice Site Mutations in *ATP7A*. No Activity of *ATP7A* Splice Variants Missing Exon 10 or Exon 15

**DOI:** 10.3389/fnmol.2021.532291

**Published:** 2021-04-21

**Authors:** Lisbeth Birk Møller, Mie Mogensen, David D. Weaver, Per Amstrup Pedersen

**Affiliations:** ^1^Department of Clinical Genetics, Applied Human Molecular Genetics, Kennedy Center, Copenhagen University Hospital, Glostrup, Denmark; ^2^Department of Medical and Molecular Genetics, Indiana University School of Medicine, Indianapolis, IN, United States; ^3^Department of Biology, University of Copenhagen, Copenhagen, Denmark

**Keywords:** *ATP7A*, Menkes disease, occipital horn syndrome, genotype-phenotype, splice site mutations, splice-variant

## Abstract

Disease-causing variants in *ATP7A* lead to two different phenotypes associated with copper deficiency; a lethal form called Menkes disease (MD), leading to early death, and a much milder form called occipital horn syndrome (OHS). Some investigators have proposed that an *ATP7A* transcript missing exon 10 leads to a partly active protein product resulting in the OHS phenotype. Here, we describe an individual with OHS, a biology professor, who survived until age 62 despite a splice site mutation, leading to skipping of exon 15. *ATP7A* transcripts missing exon 10, or exon 15 preserve the reading frame, but it is unknown if either of these alternative transcripts encode functional protein variants. We have investigated the molecular consequence of splice site mutations leading to skipping of exon 10 or exon 15 which have been identified in individuals with OHS, or MD. By comparing *ATP7A* expression in fibroblasts from three individuals with OHS (OHS-fibroblasts) to *ATP7A* expression in fibroblasts from two individuals with MD (MD-fibroblasts), we demonstrate that transcripts missing either exon 10 or exon 15 were present in similar amounts in OHS-fibroblasts and MD-fibroblasts. No ATP7A protein encoded from these transcripts could be detected in the OHS and MD fibroblast. These results, combined with the observation that constructs encoding *ATP7A* cDNA sequences missing either exon 10, or exon 15 were unable to complement the high iron requirement of the *ccc2*Δ *yeast* strain, provide evidence that neither a transcript missing exon 10 nor a transcript missing exon 15 results in functional ATP7A protein. In contrast, higher amounts of wild-type *ATP7A* transcript were present in the OHS-fibroblasts compared with the MD-fibroblasts. We found that the MD-fibroblasts contained between 0 and 0.5% of wild-type *ATP7A* transcript, whereas the OHS-fibroblasts contained between 3 and 5% wild-type transcripts compared with the control fibroblasts. In summary these results indicate that protein variants encoded by *ATP7A* transcripts missing either exon 10 or exon 15 are not functional and not responsible for the OHS phenotype. In contrast, expression of only 3-5% of wild-type transcript compared with the controls permits the OHS phenotype.

## Introduction

*ATP7A*, an X-linked gene, encodes a copper-transporting ATPase that ensures the ATP-driven translocation of copper ions across cellular membranes (Skjørringe et al., 2017). The gene is responsible both for copper loading of several copper-requiring enzymes and for efflux of copper from the cell (Kaler, 2016). At low and at normal physiological copper concentrations, the gene product, ATP7A, is located in the trans-Golgi network (TGN) where copper loading of enzymes in the secretory pathway takes place. At increased cellular copper concentration, the ATP7A protein is translocated to the plasma membrane, probably to excrete copper from the cell (Jensen et al., 1999; Skjørringe et al., 2017). Pathogenic variants in *ATP7A* result in three clinically distinct X-linked inherited disorders: Menkes disease (MD; OMIM #309400), occipital horn syndrome (OHS; OMIM #304150), and distal motor neuropathy spinal muscular atrophy [SMAX3, OMIM #X-linked distal spinal muscular atrophy-3 (SMAX3) X-linked distal spinal muscular atrophy-3 (SMAX3) X-linked distal spinal muscular atrophy-3 (SMAX3) X-linked distal spinal muscular atrophy-3 (SMAX3) 300489]. Only MD and OHS are copper-deficiency syndromes (Kaler, 2016). The severe, classic form of MD is characterized by progressive neurodegeneration, connective tissue abnormalities, distinctive “kinky” hair, and ultimately death in early childhood (Møller et al., 2011; Kaler, 2016). On the other hand, the OHS is milder than MD and characterized by connective tissue manifestations such as mild skin laxity, hernias, bladder diverticula, varicosities, and skeletal abnormalities including occipital exostoses, the finding giving rise to the syndrome’s name (Kaler, 2016). Thus, the copper defect leads to a lethal MD phenotype and to a relatively mild OHS phenotype, respectively.

Occipital horn syndrome has been reported in relatively few individuals. To date, 375 pathogenic variants in the *ATP7A* gene are registered in the Human Mutation Database^1^. A total of 18 variants identified in individuals with OHS have been published (Table 1).

**TABLE 1:** Published variants identified in individuals with OHS (18 different mutations).

*ATP7A* variant	Location	Proposed major effect on *ATP7A* mRNA or protein	References
c.-684_c-587del98bp	Promoter	Regulation of transcription. No detectable reduction in *ATP7A* transcript observed	Levinson et al., 1996
Ex3–4 dup	Exon 3-Exon 4	Duplication exon 3–4 (frameshift) Small amount of wt transcript	Mogensen et al., 2011
c.1707+6_1707+ 9delTAAG	IVS6 donor site	Skipping of exon 6 (frameshift) Small amount of wt transcript (2–5%) compared to control	Møller et al., 2000; Skjørringe et al., 2011
c.1910C>T	Exon 8	p.(Ser637Leu) Skipping of exon 8 Correct spliced transcript	Ronce et al., 1997; Beyens et al., 2019
c.2406+3A>T	IVS10 donor site	Skipping of exon 10 (in frame)	Qi and Byers, 1998; Skjørringe et al., 2011; This study
c.2407-433A>G	Intron 10	Insertion of 98 bp (pseudoexon, frameshift) between exons 10 and 11 Small amount of wt transcript (13%) compared to control	Yasmeen et al., 2014;
c.2497A>G	Exon 11	p.(Ser833Gly) Skipping of exon 11 (frameshift) Correct spliced transcript (36%)	Kaler et al., 1994; Skjørringe et al., 2011
c.2771A>G	Exon 13	p.(Gln924Arg) Copper dependent trafficking preserved Show some activity in yeast complementation analysis but only after 80 h	Møller et al., 2005; Skjørringe et al., 2017
c.2917-4A>G	IVS14 acceptor site	Skipping of exon 15 (in frame)	Das et al., 1995; Skjørringe et al., 2011; Beyens et al., 2019; This study
c.3111+4A>C	IVS15 donor site	Skipping of exon 15 (in frame)	Skjørringe et al., 2011; This study
c.3293A>C	Exon 16	p. (Gln1098Pro) Correct spliced transcript	Moizard et al., 2011
c.3294+763C>G	Intron 16	Insertion of 68 bp (pseudoexon, frameshift) between exon 16 and exon 17 Small amount of wt transcript (2%) compared to control	Yasmeen et al., 2014
c.3511+5G>A	IVS17 donor site	Skipping of exon 17 (frameshift) Small amount of wt transcript	Das et al., 1995; Skjørringe et al., 2011
c.3911A>G	Exon 20	p. (Asn1304Ser) Show approximately 33% activity of normal in yeast complementation analysis	Tang et al., 2006; Kaler et al., 2008; Vonk et al., 2012
c.3974C>T	Exon 20	p.(Ala1325Val) Both OHS and MD Compromised copper dependent trafficking Shows some activity in yeast complementation analysis	Møller et al., 2005; Skjørringe et al., 2017
c.4085C>A	Exon 21	p.(Ala1362Asp) Compromised copper dependent trafficking Shows some activity in yeast complementation analysis More transcript (approximately 245%) compared to controls	Møller et al., 2005; Skjørringe et al., 2017; Donsante et al., 2007
c.4222A>T	Exon 22	p.(Lys1408*)	Bonati et al., 2017
c.4352delG	Exon 23	p.(Gly1451Valfs*14) Frameshift the last 51 aa is missing and 13 aberrant aa is attached	Dagenais et al., 2001

*aa, amino acid; bp, base pair; IVS intervening sequence; MD, Menkes disease; OHS, occipital horn syndrome; wt, wild-type.*

Splice site mutations have been found frequently in these patients with OHS (Table 1). These splice site mutations can lead to skipping of one or several exons, and the resulting transcript can preserve or alter the reading frame. So far, two different types of splice site mutations, preserving the reading frame, have been identified in patients with OHS: splice site mutations leading to skipping of exon 10 and splice site mutations leading to skipping of exon 15. Specifically, the splice site mutation c.2406+3A>T, found in our patient P1, leads to skipping of exon 10, while the other two splice site mutations, c.2917-4A>G and c.3111+4A>C in our patients P2 and P3, respectively, lead to skipping of exon 15 (Table 2). All three patients have OHS.

**TABLE 2:** *ATP7A* variants investigated in this study.

Patient	Phenotype	*ATP7A* variant	*In silico* prediction, predicted change
**Patients with variants leading to skipping of exon 10**			
P1 (USA) (94207; M0543137H)	OHS	c.2406+3A> T (IVS10, donor site) Skipping of exon 10 (in frame)	MaxEnt: −62.6% (value reduced from 9.1 to 3.4). NNSPLICE: −76.6% (value reduced from 0.93 to 0 and c.2406 + 5 reduced from 1 to 0.8). SSF: −12% (value reduced from 82.5 to 72.6). This study: ∼3% wild-type *ATP7A* mRNA.
P4 (9,129; M77 3,003H)	MD	c.2173-1G>C (IVS9, acceptor site) Skipping of exon 10 (in frame)	MaxEnt: −100% (value reduced from 8.4 to 0). NNSPLICE: −100% (value reduced from 0.86 to 0). SSF: −100% (value reduced from 85.9 to 0). This study: ∼0.5% wild-type *ATP7A* mRNA.
**Patients with variants leading to skipping of exon 15**			
P2 (USA) (9,4209; M927888H)	OHS	c.2917-4A>G (IVS14, acceptor site) Skipping of exon 15 (in frame)	MaxEnt: −4.8% (value reduced from 8.5 to 8.1). NNSPLICE: −8.9% (value reduced from 0.9 to 0.8). SSF: 0.0% (value unchanged 84.23). This study: ∼5% wild-type *ATP7A* mRNA.
P3 (USA) (94,211;B95 20,145H)	OHS	c.3111+4A>C (IVS15, donor site) Skipping of exon 15 (in frame)	MaxEnt: −7.4% (value changed from 10.5 to 9.8). NNSPLICE: −0.9% (value reduced from 1.00 to 0.99).SSF: −11.4% (value reduced from 94.4 to 83.6). This study: ∼5% wild-type *ATP7A* mRNA.
P5 (GB) (95,286; M01 47,824H)	MD	Del exon 15 Skipping of exon 15 (in frame)	This study: 0% wild-type *ATP7A* mRNA.

*MD, Menkes disease; OHS, occipital horn syndrome; P1 through P5, patients 1 through 5.*

In 1998, Qi and Byers demonstrated that exon 10 of *ATP7A* encodes a TGN signal. It was demonstrated that an *ATP7A* mRNA transcript without exon 10 encodes an ATP7A-protein variant, which is located in the endoplasmic reticulum instead of its correct location in the TGN. These authors proposed that this “mis-localized” protein may retain some copper-transporting function, resulting in the less severe OHS phenotype (Qi and Byers, 1998).

Exon 15 of *ATP7A* encodes for the CPC channel, and we have previously demonstrated (Skjørringe et al., 2017) that a mutation of this motif (p.Cys1000Arg) hampers copper-dependent translocation, leading to permanent localization into the TGN.

To evaluate if the OHS phenotype, observed in patients P1, P2, and P3, could be explained by partial activity of the protein products encoded by transcripts missing exon 10 as proposed previously (Qi and Byers, 1998) or by transcripts missing exon 15, we here investigated the molecular effects of selected mutations affecting skipping of exon 10 and exon 15 in detail.

## Materials and Methods

### Cell Cultures

Skin samples were collected from the patients, and fibroblast cultures were established. The fibroblasts were cultured in a 1:1 mixture of RPMI 1640 with 20 mM HEPES and a nutrient mixture of F-10 Ham’s medium, supplemented with 7.5% Amnio Max (Life Technologies), C100 supplement, 4% fetal calf serum, penicillin, and streptomycin.

### RNA Isolation and Characterization

Total RNA was isolated from approximately 5 × 10^6^ cells of cultured skin fibroblasts. The kit RNeasy (QIAgen, Bothell, WA) was used. Single-stranded cDNA was synthesized with the High-Capacity cDNA Archive Kit in accordance with the manufacturer’s instructions (Applied Biosystems, Foster, CA). For 50 μl of cDNA suspension, 0.5 μg RNA was used. The cDNA was used for PCR amplification with *ATP7A*-specific primer pairs flanking the mutation of interest. For PCR amplification of the region from exon 8 to exon 12, the primers 8F: aggttttgaagcttctttggtcaag and 12R: ctggaaatttgcctcctggaact were used. For PCR amplification of the region from exon 13 to exon 17, the primers 13F: aacgggtcactgcttatctgcg and 17R: gtcctctatattccagttattc were used. The PCR products were separated on a 1% agarose gel, and the fragments were excised and purified using Qiaquick gel extraction kit (Qiagen) before sequencing with the PCR amplification primers. For fragment separation by capillary electrophoresis using an ABI3730, 5′ FAM-labeled 12R and 17R primers were used in combination with 8F and 13F, respectively. In both cases, the enzyme AccuPrime DNA Polymerases (Invitrogen) was used, and PCR was performed using 35 cycles. *ATP7A* reference sequences are NM_000052.6 and NG_013224.2.

### Sanger Sequencing

The Big Dye Terminator v.3.1 Cycle Sequencing Kit was used for sequencing, and the products were analyzed in an ABI model 310 capillary sequencer.

### Quantitative RT-PCR

Real-time PCR was performed for relative quantification of *ATP7A* transcript. The predesigned *ATP7A* probe Hs00921957_m1 (recognizing the boundaries between exon 14 and exon 15), and the predesigned *ATP7A* probe Hs00921963_m1 (recognizing the boundaries between exon 1 and exon 2), were used. *ATP7A* probes recognizing exon 10–exon 11 boundaries, exon 9–exon 11 boundaries and exon 14–exon 16 boundaries in *ATP7A* mRNA were designed specifically for this project. All *ATP7A* probes were 6-carboxy-fluorescein (FAM) labeled. A FAM or VIC (Applied biosystems proprietary dye)-labeledprobe and primers for the human *GAPDH* transcript (part numbers 4352934E and 4326317E, respectively) were used as an endogenous control. Relative quantification of *GAPDH* transcript was carried out on parallel samples. All probes were purchased from Applied Biosystems. Standard curves of (C_*T*_) values compared with log cDNA concentration were prepared by assaying fivefold serial dilutions of control cDNA, from 100 to 0.16 ng/sample (assuming that the cDNA concentration is identical to the mRNA concentration used for cDNA preparation) with the *GAPDH* and *ATP7A* probes, respectively.

### SDS-PAGE and Western Blotting (WB)

Cultured patient fibroblasts were harvested by trypsination and lysed in lysis buffer (50 mM Hepes pH 7.6; 250 mM NaCl; 0.1% NP40; 5 mM EDTA) for 30 min on ice. The SDS page and WB were performed as described previously (Skjørringe et al., 2017). The chicken polyclonal anti-ATP7A (ab13995, Abcam; 1:1,000), recognizing the C-terminus of the ATP7A protein, and the rabbit polyclonal anti-GAPDH (NB300-327, Novus biological; 1:5,000) were used as primary antibodies. Donkey anti-chicken (HRP) (DAKO)^2^ and swine anti-rabbit (HRP) (DAKO)^3^ to target the primary antibodies against ATP7A and GADPH, respectively, were used as secondary antibodies.

### Plasmid Constructions

Plasmid pPAP6168 expressing wild-type human ATP7A was created by *in vivo* homologous recombination in *S. cerevisiae* strain PAP6094 between *Sal*I-, *Hin*dIII-, and *Bam*HI-digested pEMBLyex4 (Cesareni and Murray, 1987) and PCR-amplified Menkes cDNA with 35 nucleotides 5’ and 3’ extensions identical to the sequences flanking the multicloning site in pEMBLyex4. Plasmids pPAP7229 (missing exon10) and pPAP7232 (missing exon 15) were generated by *in vivo* homologous recombination between *Sal*I-, *Hin*dIII-, and *Bam*HI-digested pEMBLyex4 and two PCR fragments encompassing either exons 1–9 and exons 11–23 or exons 1–14 and exons 16–23. In-frame C-terminal tagging of wild-type, exon 10 and exon 15 missing *ATP7A* with yEGFPs (Cormack et al., 1997) was constructed by *in vivo* homologous recombination in PAP6094 between *Sal* I-, *Bam* HI-, and *Hin*dIII-digested pEMBLyex4 expression vector (Cesareni and Murray, 1987) and each of the three ATP7A cDNA fragments and a GFP PCR fragment amplified with primers adding an N-terminal TEV (Tobacco Etch Virus) proteolytic site and a C-terminal HIS_8_ tag. Nucleotide sequences were confirmed by DNA sequencing.

### Yeast Strain

*S. cerevisiae* strain PAP6094 used for complementation assays was derived from Y1369 (Euroscarf) (*Mat*α; *his3Δ1*; *leu2Δ0*; *met15Δ0*; *URA3Δ0*; *ccc2: kanMx4*) by integration of a *UPR-LacZ* reporter plasmid into the *his3* locus by homologous recombination (Jørgensen and Pedersen, 2001).

### Live Cell Bioimaging of Yeast Cells

Fluorescence was imaged in live *ccc2Δ* PAP6094 cells that had been induced for expression of the GFP-tagged Menkes alleles for 18 h at 30°C. Images were captured using an Optronics Magnafire model S99802 camera coupled to a Nikon Eclipse E600 microscope at a 1,000 × magnification.

### Complementation Assay

Complementation assays were performed as described by Forbes and Cox (1998). Shortly, 3, 5, or 7 μl of cells grown in glucose minimal medium to OD_450_ = 0.5 were spotted on minimal medium containing 150 μM Fe (NH_4_)SO_4_, 1 μM CuSO_4_, 1 mM ferrozine, and 2% galactose as carbon source. Cells were incubated at 30°C.

### Patients

P1 has previously been described in 1998 (Qi and Byers, 1998). The patient was at that time 29 years old with typical manifestations of OHS. He was from a family with an identified *ATP7A* mutation, c.2406 + 3A > T, in four generations. His clinical findings included occipital exostoses and recurrent hernias. Since late childhood, he also had experienced orthostatic hypotension, had 5–10 stools per day, and needed periodic catheterization due to atonic bladder.

P2 has previously been described in 1995 (Das et al., 1995). At that time, he was 14 years old with findings of cutis laxa and muscle wasting. He also has had musculoskeletal abnormalities and multiple compression fractures of his vertebrae, which has required treatment. The patient also suffered from recurrent bladder rupture and atonic bladder, the latter requiring intermittent catheterization. In the perinatal period, he had mild hypotonia and radiographically cranial contour abnormalities, and wormian bones were observed. Radiographs at age 14 years revealed the characteristic occipital horns found in OHS. Cognition was normal.

Previously, P3 has been described (Hollister, 1982; Sartoris et al., 1984; Hennekan et al., 2010), but we update his history here. Informed consent was obtained from the person. He was born after a full-term, uneventful gestation and delivery. No neonatal or infantile medical problems occurred. As an adult, he was a tall, slim white male with an especially narrow shoulder girdle. His height was 182 cm (85% tile) and weight around 60 kg (30% tile). For most of his adult life, he was employed as a biology professor at a United States university. He was alert and intelligent with a thin and narrow face, high forehead, and high-arched palate, long and wide neck, bilaterally palpable occipital bony spurs at the base of the skull, narrow shoulders with short and broad clavicles, large superficial varicosities of the legs, and joint hypermobility. His feet had been flat since age 6, and getting suitable footwear had been a major problem. At age 61/2, he required surgical relief for bladder neck obstruction causing retention and later for bladder diverticula. At age 11, he developed a left inguinal hernia, which was repaired at age 14. During his childhood and teenage years, he was active in multiple sports, but was generally underweight and not as strong as his peers. He died suddenly from an unknown cause at the age of 62.

**FIGURE 1:**
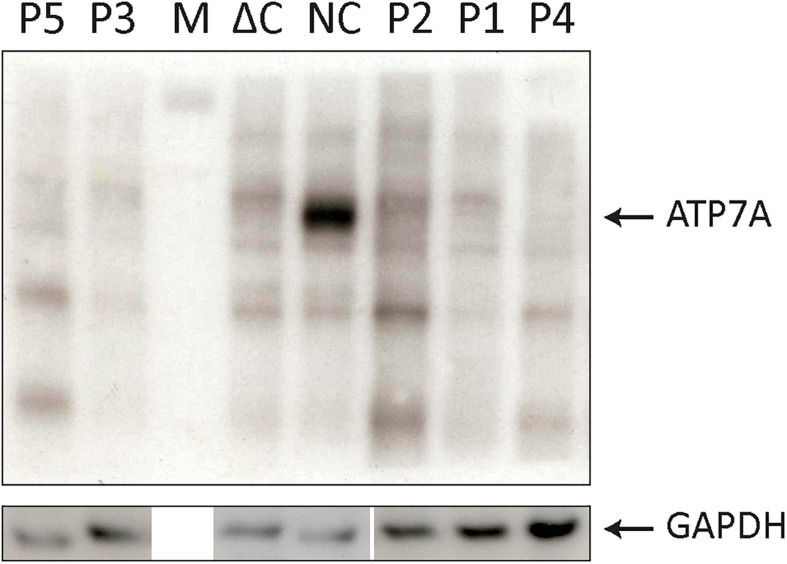
SDS-PAGE and Western blotting (WB) analysis of cell lysates from P1–P5 fibroblasts. Antibodies against ATP7A were used as indicated. Positive normal control fibroblasts (NC) and negative control fibroblasts (ΔC), from a Menkes disease (MD) patient hemizygous for deletion of the entire *ATP7A* gene were included. An intense band representing wild-type *ATP7A* was observed in NC. In all the patients, P1–P5 only bands present also in ΔC were observed. M indicates the protein ladder. WB of the GAPDH probe from similar samples was used as loading control.

Our patients P4 and P5 both were suffering from MD and were included in this study for comparison. They previously had been reported by Skjørringe et al. (2011). Furthermore, a fibroblast from a patient with MD, hemizygous for a genomic deletion of the entire *ATP7A* gene, has been included as a negative control (ΔC) in Figure 1; Skjørringe et al. (2017).

The used four control fibroblasts were obtained from our fibroblast biobank consisting of anonymized samples obtained from individuals with no identified diseases (healthy carriers, spouses, etc.).

## Results and Discussion

We investigated fibroblast cultures from three individuals with OHS (P1, P2, and P3) and from two individuals with MD (P4 and P5), all hemizygous for a pathogenic variant in the *ATP7A* gene. In addition, we included control fibroblasts from four normal individuals (NC).

P1 with OHS and P4 with MD hemizygous for splice site mutations previously shown (P1) and predicted to result in a final *ATP7A* transcript devoid of exon 10 (Table 2). P2 and P3 with OHS had splice site mutations previously and predicted to lead to skipping of exon 15. P5 with MD encoded an *ATP7A* transcript without exon 15 due to a genomic deletion of exon 15 (Table 2). *In silico* prediction of the effect of the splice site mutations using the interactive biosoftware programs MaxEnt, NNSplice, and SSF created by Alamut Visual predicted a strong effect on the splicing of the variant in P4, an intermediate effect of the variant in P1 and a relative small effect of the variants in P2 and P3 (Table 2).

**FIGURE 2:**
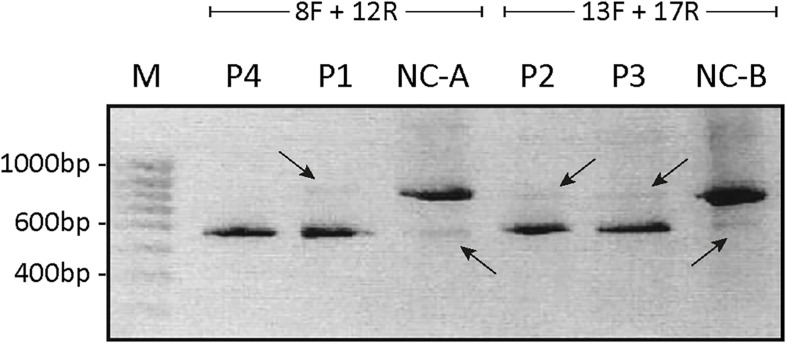
Agarose-gel of separated PCR fragments. The cDNA fragment spanning from exon 8 to exon 12 was PCR amplified from persons P1, P4, and control (NC-A) using primers 8F and 12R, and the cDNA fragment from exon 13 to exon 17 was PCR amplified from persons P2, P3, P5, and control (NC-B) using primers 13F and 17R. The PCR products were separated on a 1% agarose gel. The bands were purified and Sanger sequenced. The estimated size of the amplified fragments is indicated. A 100-bp ladder (M) was used as reference. Intense bands representing *ATP7A* mRNA without exon 10 in P1 and P4 and representing *ATP7A* without exon 15 in P2 and P3, respectively, are shown. Only very faint bands representing *ATP7A* wild-type fragments are observed in P1, P2, and P3 (indicated by arrows pointing down). In the two NC samples, intense bands representing wild-type transcripts are observed in addition to very faint bands representing *ATP7A* mRNA without exon 10 (NC-A) or without exon 15 (NC-B), respectively (indicated by arrows pointing up).

To investigate the resulting effects of the splice site mutations in P1–P4 in more detail, RNA was isolated from fibroblasts from each patient and investigated by RT-PCR, using primers spanning the suspected skipped exon. To determine the effects of the splice site mutations located in the acceptor site in intron 9 in P1 and in the donor site in intron 10 in P4, primers spanning the cDNA sequences from exon 8 to exon 12 were used. To determine the effect of the splice site mutations located in the acceptor site in intron 14 in P2 and in the donor site in intron 15 in P3, primers spanning the cDNA sequence from exon 13 to exon 18 were used. The products were separated on a 1% agarose gel (Figure 2).

As expected, the major band in both P1 and P4 was approximately 500 bp compatible with a product devoid of exon 10, whereas the major product in the control sample was approximately 700 bp. Previously, Qi and Byers (1998) published that only a single *ATP7A* transcript, a transcript without exon 10, had been observed in P1 by RT-PCR investigation. However, in the present experiment, a faint band of similar size as found in the wild-type control sample, was identified in P1, whereas no band of this size was observed in P4. Also, in the control sample NC-A, there was a faint band of approximately 500 bp (Figure 2). Sanger sequencing of the two bands in P1 confirmed that, in addition to the major band representing *ATP7A* transcript without exon 10, there was a small amount of the wild-type transcript containing exon 10. We also found that, in addition to the major band representing *ATP7A* transcript containing exon 10, the control sample, NC-A, also expresses a transcript without exon 10. It has previously been demonstrated by Qi and Byers (1998) that a transcript lacking exon 10 constituted approximately 10% of *ATP7A* transcripts in normal fibroblasts. Exon 10 encodes transmembrane domains 3 and 4 including the TGN motif.

Similarly, the major PCR band from P2 and P3 was approximately 550 bp as would be expected for a product missing exon 15. The major band in the control sample NC-B, which contained exon 15, was approximately 750 bp. In both P2 and P3, small amounts of the wild-type band were also observed (Figure 2). Sanger sequencing of the products in both P2 and P3 confirmed that the major band in each represents a product without exon 15, whereas the minor band in each represents the wild-type product containing exon 15. In the control sample NC-B, the major band represents, as expected, wild-type product containing exon 15. Furthermore, the minor band in the control sample NC-B represents an *ATP7A* transcript without exon 15. Exon 15 codes for the sixth transmembrane domain including the critical CPC motif common for all heavy metal-transporting ATPases.

**FIGURE 3:**
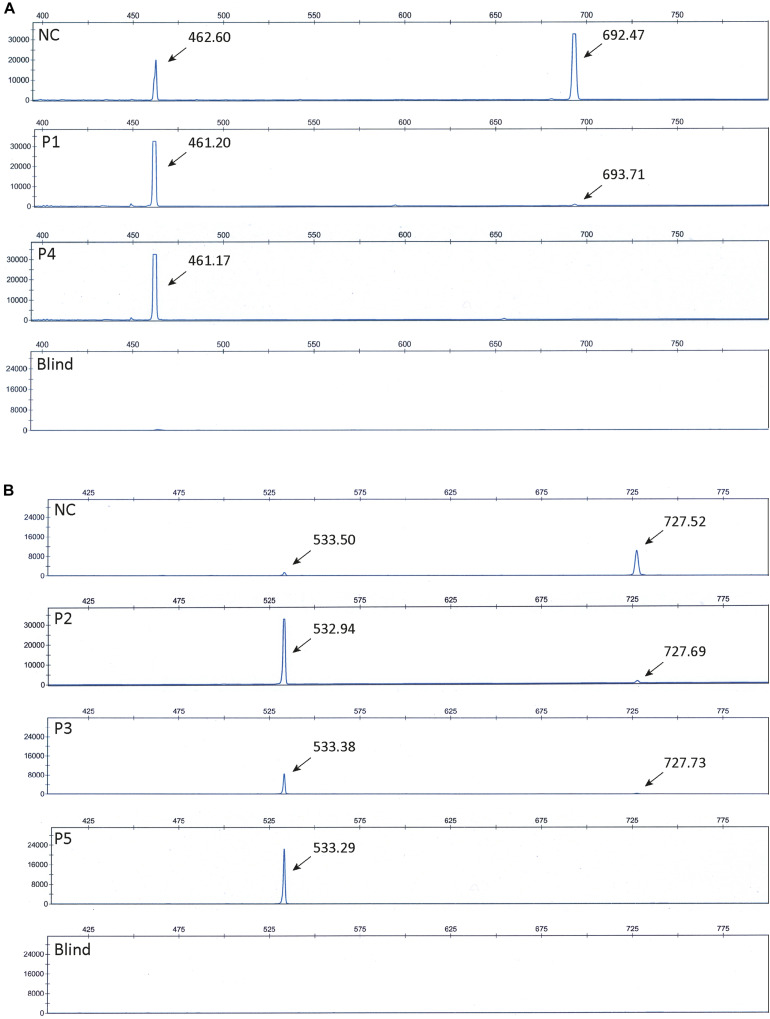
ABI files of separated Fam-labeled PCR fragments. **(A)** Investigation of fragments obtained by PCR amplification of cDNA generated from P1, P4, and normal control (NC) using the primer-pair 8F and 5′ Fam labeled 12R. Fragments representing normal spliced *ATP7A* mRNA (692-693 bp) and fragments representing *ATP7A* mRNA without exon 10 (461-462 bp) were observed as indicated. **(B)** Investigation of fragments obtained by PCR amplification of cDNA generated from P2, P3, P5, and normal control (NC) using the primer-pair 13F and 5’ Fam labeled 17R. Fragments representing normal spliced *ATP7A* mRNA (727-728 bp) and fragments representing *ATP7A* mRNA without exon 15 (533-534 bp) were observed as indicated. Blind represents a sample without cDNA added prior to PCR amplification.

To further confirm that the obtained fragments in the patient and control samples represent normal splices and products missing exon 10 and 15, respectively, we repeated the PCR amplification using Fam-labeled 5′ reverse primers and separated the fragments using ABI-3730. Using this method, fragment sizes are obtained very precisely. The ABI files of 8F/12R-amplified products (Figure 3A) confirmed that normal spliced *ATP7A* mRNA (692-693 bp) was present in P1, whereas no normal spliced product could be observed in P4, and in addition, *ATP7A* mRNA without exon 10 (461-462 bp) was present in both P1, P4 and the normal control sample (NC). Similarly, the ABI files of 13F/17R-amplified products (Figure 3B) confirmed that normal spliced *ATP7A* mRNA (727-728 bp) was present in P2, P3, and NC, but not in P5, and in addition, *ATP7A* mRNA without exon 15 (533-534 bp) was present in all samples, P2, P3, P5, and NC. As normal PCR is not quantitative, we continued our investigation using real-time PCR for quantitative determination of the different splice variants of *ATP7A* mRNAs.

To test if skipping of exon 10 or exon 15 affects the amount of the *ATP7A* transcript, we quantitated the relative amount of total *ATP7A* transcript in the patient samples. We did this by utilizing real-time PCR using a probe recognizing the boundary between exon 1 and exon 2 expected not to be affected by erroneous exon 10 and 15 splicing. For comparison, we included mRNA from four different controls. The amount of *ATP7A* transcript did not differ significantly in any of the patients compared with the controls (Figure 1), indicating that transcripts without exon 10 or exon 15 were not target for nonsense-mediated decay (Maquat, 1995).

Furthermore, to quantitate the amount of wild-type transcript, we performed real-time RT-PCR using TaqMan probes, which specifically recognize the wild-type transcript. For detection of the wild-type transcript in P1 and P4, a probe that recognized the boundary between exons 10 and 11 was used. For detection of the wild-type transcript in each of P2, P3, and P5, we used a probe recognizing the boundary between exons 14 and 15.

The amount of wild-type transcript was significantly reduced in P4 compared with P1. The results revealed that approximately 3% of wild-type *ATP7A* transcript was present in P1, whereas about 0.5% wild-type transcript was present in P4, when compared with the normal controls. For both P2 and P3, we found approximately 5% wild-type *ATP7A* and no wild-type transcript in P5 (0%, as expected from a genomic deletion of exon 15) (Figure 1). Thus, higher amounts of wild-type transcript were present in the OHS-fibroblasts, P1, P2, and P3 compared with the MD-fibroblasts P4 and P5. Interestingly, the amount of wild-type transcript followed the same order as the predicted severity of the pathogenic variants on splicing, but the observed splicing effects of variants in P2 and P3 were more severe than predicted (Table 2). Thus, while the *in silico* predictions for variants affecting splicing of exon 10 were consistent with the obtained real-time RT-PCR data, this was not the case for variants affecting splicing of exon 15.

To quantify the amount of transcript without exon 10 in P1 and P4, compared with the controls, we performed RT-PCR using a TaqMan probe that recognizes the boundaries between exon 9 and exon 11, which only is present in exon 10-skipped transcript. The analysis revealed that the amount of exon 10-skipped product in P1 and P4 were significantly increased compared with that in the controls, but the amount in P1 did not differ significantly from the amount in P4. The amount of exon 10-skipped product in the control fibroblast was approximately 6.5% of the amount in P1 and P4 (Figure 1).

Furthermore, we quantitated the amount of transcript without exon 15 in P2 and P3 compared with P5 by real-time RT-PCR using a TaqMan probe that recognized the boundaries between exon 14 and exon 16, which only is present in exon 15-skipped transcripts. The analyses revealed that the amount of exon 15-skipped product in P2 and P3 with OHS was not significantly different from the amount present in P5 with MD. The amount of exon 15-skipped product in the control fibroblast was between 7% and 14% of the amount in P2, P3, and P5 (Figure 1).

Thus, the amounts of exon 10- or exon 15-skipped transcripts were not significantly higher in OHS-fibroblasts compared with those in MD-fibroblasts, making it unlikely that any of these transcripts contribute to the variability of the severity of the disease.

The normal ATP7A protein is expected to transfer copper from the cytosol into the lumen of TGN and, in this way, supplies copper-requiring enzymes, produced in the secretory pathway, with copper (Schmidt et al., 2018). However, it is possible that copper loading also takes place in other cellular compartments. Exon 10-skipped transcripts have been demonstrated to encode an ATP7A protein, with a slightly lower molecular weight and location in the endoplasmic reticulum (ER), due to the lack of a Golgi localization signal (Francis et al., 1998). ATP7A protein encoded by exon 10-skipped transcript has previously been suggested also to be present in ER in fibroblasts obtained from P1 (Qi and Byers, 1998). These investigators proposed that this location permits certain copper-requiring enzymes to receive copper at an earlier stage in the secretory pathway, such as ER, and in this way, this splice-variant might be partly functional, permitting the mild OHS phenotype.

**FIGURE 4:**
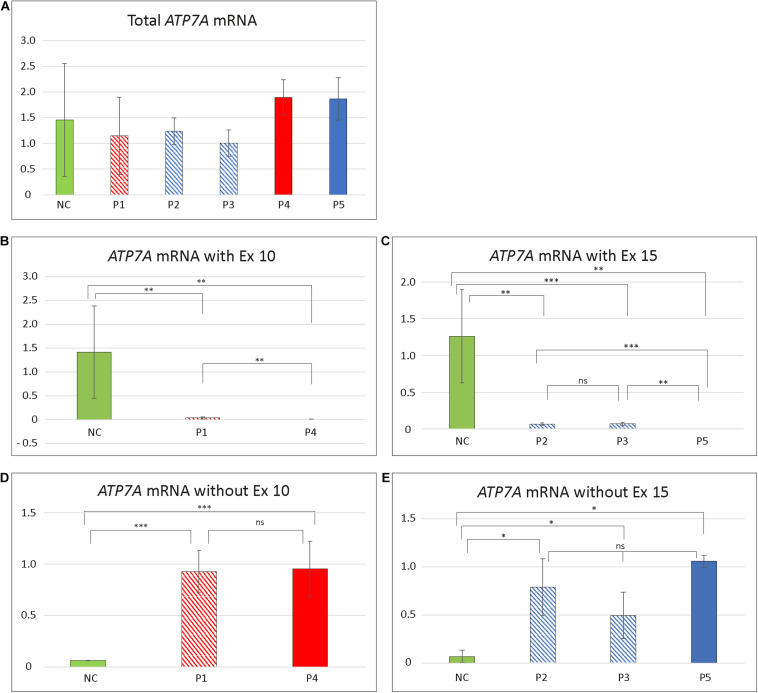
Relative amount of different ATP7A transcript in patient fibroblasts. **(A)** Total amount of transcript in the five patients compared to controls. No significant difference in amount in any of the patients compared with controls (NC) was identified (P1/NC: *p* = 0.4884; P2/NC: *p* = 0.4926; P3/NC: *p* = 0.2703; P4/NC: *p* = 0.4156; P5/NC: *p* = 0.5293). **(B)** Total amount of exon 10 (Ex10) containing transcript in P1 and P4. The amount of Ex10 containing transcript was significantly reduced compared to controls in both patients (P1/NC: *p* = 0.0026; P4/NC: *p* = 0.0011). Furthermore, the amount of transcript was significantly higher in P1 compared with P4 (*p* = 0.0030). **(C)** Total amount of exon 15 (Ex15) containing transcript in P2, P3, and P5. All patients had significantly reduced amount of Ex15 containing transcript compared with controls (P2/NC: *p* = 0.0038; P3/NC: *p* = 0.00025; P5/NC: *p* = 0.0029). The amount of Ex15 containing transcript was significantly higher in P2 (*p* = 0.0040) and P3 (*p* = 0.00065) compared with P5. No significant difference in amount was obtained between P2 and P3 (*p* = 0.6845). **(D)** Amount of ATP7A mRNA without Ex10. The amount of transcript without Ex10 were significantly increased in P1 and P4 compared with controls (P1/NC: *p* = 3.57 × 10^– 8^; P4/NC: *p* = 0.00034). No significant difference in amount was observed between P1 and P4 (*p* = 0.9170). **(E)** Amount of ATP7A mRNA without Ex15. The amount of transcript without Ex15 was significantly increased in P2, P3, and P5 compared with controls (P2/NC: *p* = 0.0107; P3/NC: *p* = 0.0210; P5/NC: *p* = 0.0430). No significant difference in amount was observed between P2, P3, and P5 (P2/P3: *p* = 0.1525; P2/P5: *p* = 0.6819; P3/P5: *p* = 0.2821). The relative amounts of *ATP7A* mRNA in **(A–C)** are calculated by linear regression of lines generated by standard curves and **(D,E)** calculated using the ΔΔCT method. The amount of *GAPDH* transcript was used as a normalization reference. Data obtained for P4 in **(D)** and P2 in **(E)** were pooled from two independent mRNA isolations; for all the additional experiments, data were pooled from at least three independent mRNA isolations. In all experiments, at least three different control samples were used. Student *t*-test, with significance level *p < 0.05, **p < 0.005, and ***p < 0.0005, was used. Error bars represent SEM (*n* = 2–3). NC values are illustrated as green columns. Values for variants affecting splicing of exon 10 are illustrated as shaded red (P1 with OHS) and full red (P4 with MD) columns. Values affecting splicing of exon 15 are illustrated as shaded blue (P2 and P3 with OHS) and full blue (P5 with MD) columns.

To test for expression of ATP7A proteins encoded by the exon 10- or exon 15-skipped transcripts, we analyzed cell lysates from the five fibroblast cultures (P1–P5) by SDS-PAGE and Western blotting (WB) (Figure 4). As exon 10 encodes for 78 amino acids and exon 15 for 65 amino acids, the predicted size of proteins encoded by these transcripts would be approximately 9 and 7 kDa less compared with the wild-type ATP7A protein, respectively. As expected, the ATP7A protein (165–170 kDa) was present in normal positive control fibroblasts (NC) but absent in negative control fibroblasts (ΔC), in which the entire *ATP7A* gene was deleted. Surprisingly, no ATP7A protein could be detected in any of the patient’s fibroblasts P1–P5. The small amount of wild-type transcript present in P1, P2, P3, and P4 did not result in protein that was detectable by WB. Only in bands also present in the negative control sample could ΔC be detected. A cleaner WB might have allowed detection of the protein. More surprising, although large amounts of exon 10- and exon 15-skipped *ATP7A* transcript were present in the fibroblasts of P1–P5, no ATP7A protein could be detected, indicating a reduced synthesis or reduced stability of these ATP7A protein variants, despite a similar or larger amount of protein loaded with P1–P5 compared with the normal positive control, NC.

**FIGURE 5:**
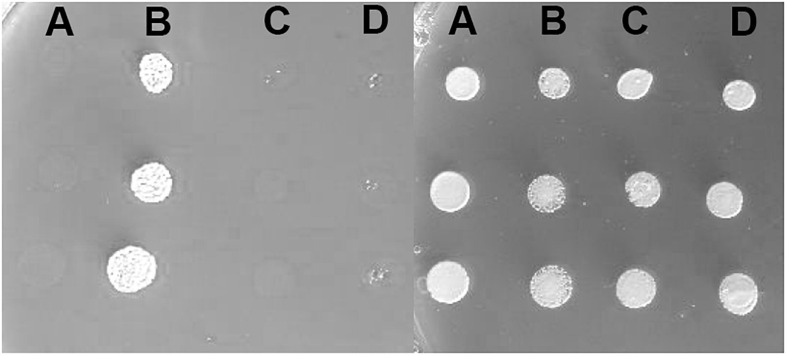
Complementation of the *ccc2*Δ iron requiring phenotype on agar plates. Left panel: The exon 10 and exon 15 missing variants were investigated for their ability to complement the high iron requirement of a *ccc2*Δ yeast strain by plating cells on agar plates containing the iron chelator ferrozine. **(A)** *ccc2*Δ yeast cells expressing no *ATP7A* (empty vector). **(B)** *ccc2*Δ yeast cells expressing wild-type *ATP7A*. **(C)** *ccc2*Δ yeast cells expressing *ATP7A* variant missing exon 10. **(D)** *ccc2*Δ yeast cells expressing *ATP7A* variant missing exon 15. Right panel: To control for cell viability, each yeast strain was also spotted on iron-containing agar plates.

Furthermore, to determine the *in vivo* activities of the two variants, we used a yeast complementation assay based on a strain lacking the single *ATP7A* ortholog, *CCC2*. The *ccc2*Δ yeast host is unable to thrive under iron-limited conditions, while growth can be rescued by the expression of wild-type *ATP7A*. Deletion of either exon 10, or exon 15, respectively, was introduced into the full-length *ATP7A* cDNA sequence. Neither a construct missing exon 10 nor a construct missing exon 15 was able to complement the high iron requirement of the *ccc2*Δ yeast strain, which further supports that the protein products encoded by these constructs do not have any Cu-ATPase activity (Figure 5).

**FIGURE 6:**
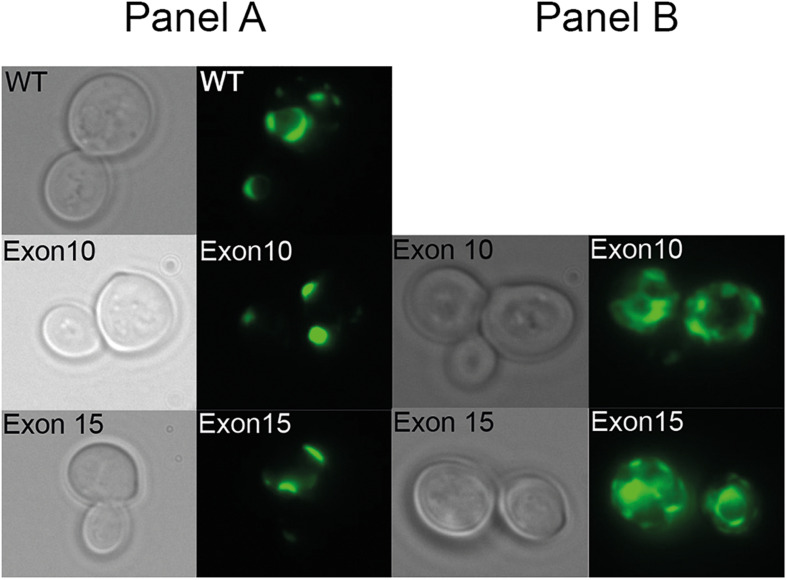
Live cell bioimaging of the ccc2Δ yeast strain PAP6094 expressing green fluorescent protein (GFP)-tagged wild-type ATP7A, exon10 or exon15 missing *ATP7A* variants. Yeast cultures were grown at 30°C in galactose minimal medium overnight prior to live cell bioimaging as described in the *Materials and Methods* section. Representative images of the two observed classes panels **(A,B)** of cells expressing *ATP7A* variants missing exon 10, or exon 15 in addition to cells expressing wild-type *ATP7A* panel **(A)** are shown. For each ATP7A variant, a phase-contrast image and the corresponding GFP image are shown.

To verify that the inability of the exon 10 and exon 15 deletion mutants to complement the high iron requirement of the *ccc2* yeast strain was not due to lack of protein accumulation, we C-terminally tagged each mutant allele and wild-type with GFP and examined their expression by live cell bioimaging. As can be seen from Figure 6, GFP fluorescence was observed in yeast cells expressing either a mutant allele or wild-type showing that each of the three proteins was produced. However, in contrast to wild-type ATP7A that showed a uniform cellular localization in the yeast population as evident from Figure 6, localization of the two exons lacking variants revealed a more heterogenous localization. Some cells revealed a wild-type-like localization (panel A) while others showed a more diffused localization (panel B).

In summary, only the amount of wild-type transcript was higher in patients with OHS compared with patients with MD, whereas no significant differences were observed in the amount of exon 10- or exon 15-skipped transcripts in the two types of patients. Furthermore, ATP7A constructs missing either exon 10 or exon 15 failed to show any complementation of a *ccc2*Δ yeast strain. Based on these observations, we hypothesize that neither *ATP7A* variants encoded by exon 10 deleted nor exon 15 deleted transcripts exhibit any copper transport activity.

We suggest that the OHS phenotype occurred in P1, P2, and P3 in contrast to MD in P4 and P5 is due to the presence of small amounts of wild-type ATP7A transcript. We have previously indicated that between 2 and 5% of the amount compared with the control fibroblast is enough to permit the milder OHS phenotype (Møller et al., 2009; Møller, 2015). In agreement with this hypothesis, we observed that fibroblasts from individuals with OHS (P1, P2, and P3) accumulate between 3 and 5% wild-type ATP7A transcript, whereas fibroblasts from individuals with MD (P4, P5) only accumulate between 0 and 0.5% wild-type *ATP7A* transcript. We do not, however, have any explanation for the unusually mild phenotype in P3 compared with those in P1 and P2. One possibility is, however, that the amount of wild-type *ATP7A* transcript differs in different organs between the three OHS patients, and this explains the observed phenotypic differences.

## Data Availability Statement

The original contributions presented in the study are included in the article/Supplementary Material, further inquiries can be directed to the corresponding author/s.

## Ethics Statement

The studies involving human participants were reviewed and approved by National Videnskabsetisk Komite (www.nvk.dk). Written informed consent to participate in this study was provided by the participants’ legal guardian/next of kin.

## Author Contributions

LM, MM, DW, and PP drafted the work and made substantial contributions to the conception and design of the work. DW provided clinical information regarding P3. MM performed the RT-PCR. MM and LM performed the real-time RT-PCR. PP performed the yeast-based experiments. All authors contributed to the article and approved the submitted version.

## Conflict of Interest

The authors declare that the research was conducted in the absence of any commercial or financial relationships that could be construed as a potential conflict of interest.

## Footnotes

1 http://www.hgmd.org

2 www.dako.com/dk/

3 www.dako.com/dk/
