# Supplementary material for: Occipital Horn Syndrome as a Result of Splice Site Mutations in ATP7A. No Activity of ATP7A Splice Variants Missing Exon 10 or Exon 15
Source: Front Mol Neurosci. 2021 Apr 21;14:532291. doi: 10.3389/fnmol.2021.532291 (PMC8097048; doi:10.3389/fnmol.2021.532291)
Supplement: Supplementary file 1 [file Data_Sheet_1.PDF]

MNK 407 P1

PCR product: 8U og 12L

Sekventeret: ex8 - ex9 - ex11 - ex12

Primer 8U:**ex8** - ex9 - **ex11** - ex12

```
Score =  335 bits (174),  Expect = 1e-88
 Identities = 216/232 (93%), Gaps = 3/232 (1%)
 Strand=Plus/Plus

Query  33    ACAATGGAGACGGTCTCCNCNCGTGAGTCTGTCCCNCTGTAANCCTGTAATGGGGCTGAT  92
             ||||||||||||||||   |  ||||||||||    |||||  |||||||||||||||||
Sbjct  2089  ACAATGGAGACGGTCTTTTCTTGTGAGTCTGTTTTTCTGTATTCCTGTAATGGGGCTGAT  2148

Query  93    GATATATATGATGGTTTATGGACCACCACTTTTGCAACTCTTCACCATANTCAAAACATG  152
             || ||||||||||||| ||||||||||||||| |||||||||||||||| ||||||||||
Sbjct  2149  GACATATATGATGGTT-ATGGACCACCACTTT-GCAACTCTTCACCATAATCAAAACATG  2206

Query  153   AGTAAAGAAGAAATGATCAACCTTCATTTCTTCTATGTTCCTGGAGCGCCAGATTCTTCC  212
             |||||||||||||||||||||||||||| |||||||||||||||||||||||||||||||
Sbjct  2207  AGTAAAGAAGAAATGATCAACCTTCATT-CTTCTATGTTCCTGGAGCGCCAGATTCTTCC  2265

Query  213   AGGATTGTCTGTTATGAATTTGCTGTCCTTTTTATTGTGTGTACCTGTACAG  264
             ||||||||||||||||||||||||||||||||||||||||||||||||||||
Sbjct  2266  AGGATTGTCTGTTATGAATTTGCTGTCCTTTTTATTGTGTGTACCTGTACAG  2317


Score =  283 bits (147),  Expect = 5e-73
 Identities = 170/184 (92%), Gaps = 1/184 (0%)
 Strand=Plus/Plus

Query  263   AGGGCAAAACATCANAGGCTCTTGCAAAGTTATTTTCACTACAAGCTACAGAAGCAACTA  322
             |||||||||||||| ||||||||||||||||| |||||||||||||||||||||||||||
Sbjct  2550  AGGGCAAAACATCAGAGGCTCTTGCAAAGTTAATTTCACTACAAGCTACAGAAGCAACTA  2609

Query  323   TTGTAACTCTTGATTCTGATAATATCCTCCTCANTGAANAACAAGTGGATGTGGAACTTG  382
             ||||||||||||||||||||||||||||||||| |||| |||||||||||||||||||||
Sbjct  2610  TTGTAACTCTTGATTCTGATAATATCCTCCTCAGTGAAGAACAAGTGGATGTGGAACTTG  2669

Query  383   TACAACGTGNAAATATCATTAAAGTANTTCC-GGANGCAAATTTCCNGTGNATGNNCCTG  441
             ||||||||| | |||||||||||||| |||| ||| |||||||||| ||| |||  | ||
Sbjct  2670  TACAACGTGGAGATATCATTAAAGTAGTTCCAGGAGGCAAATTTCCAGTGGATGGTCGTG  2729

Query  442   TTAT  445
             ||||
Sbjct  2730  TTAT  2733
```

Primer 12L: **ex12** - ex11 - **ex9** - ex8

```
Score =  191 bits (99),  Expect = 3e-45
 Identities = 113/119 (94%), Gaps = 1/119 (0%)
 Strand=Plus/Minus

Query  22    AAGTTCCNCATCC-CTTGTTCTTCACTGAGGAGGATATTATCAGAATCACGAGTTACAGT  80
             ||||||| ||||| ||||||||||||||||||||||||||||||||||| |||||||| |
Sbjct  2668  AAGTTCCACATCCACTTGTTCTTCACTGAGGAGGATATTATCAGAATCAAGAGTTACAAT  2609

Query  81    AGTTGCTTCTGTAGCTTGTAGTGAAATTANCTTTGCANGAGCCTCTGATGTTTTGCCCT  139
             ||||||||||||||||||||||||||||| ||||||| |||||||||||||||||||||
Sbjct  2608  AGTTGCTTCTGTAGCTTGTAGTGAAATTAACTTTGCAAGAGCCTCTGATGTTTTGCCCT  2550


Score =  473 bits (246),  Expect = 2e-130
 Identities = 284/295 (96%), Gaps = 1/295 (0%)
 Strand=Plus/Minus

Query  138   CTGTACAGGTACACACAATAAAAAGGACAGCAAATTCATAACAGACAATCCTGGAAGAAT  197
             ||||||||||||||||||||||||||||||||||||||||||||||||||||||||||||
Sbjct  2317  CTGTACAGGTACACACAATAAAAAGGACAGCAAATTCATAACAGACAATCCTGGAAGAAT  2258

Query  198   CTGGCGCTCCAGGAACATAGAAGAATGAAGGTTGATCATTTCTTCTTTACTCATGTTTTG  257
             ||||||||||||||||||||||||||||||||||||||||||||||||||||||||||||
Sbjct  2257  CTGGCGCTCCAGGAACATAGAAGAATGAAGGTTGATCATTTCTTCTTTACTCATGTTTTG  2198

Query  258   ATTATGGTGAAGAGTTGCAAAGTGGTGGTCCATAACCATCATATATATCATCAGCCCCAT  317
             |||||||||||||||||||||||||||||||||||||||||||||| |||||||||||||
Sbjct  2197  ATTATGGTGAAGAGTTGCAAAGTGGTGGTCCATAACCATCATATATGTCATCAGCCCCAT  2138

Query  318   TACNGNGGAAAAAAAAAAAAACACTCACAAGAAAAGACCGTCTCCATTGTCTTATTTCTC  377
             ||| | | | | | ||||| | ||||||||||||||||||||||||||||||||||||||
Sbjct  2137  TACAG-GAATACAGAAAAACAGACTCACAAGAAAAGACCGTCTCCATTGTCTTATTTCTC  2079

Query  378   GTTTATGANCTAAGTGACTTGCTGACCGATCCTTCTTGACCAAAGAANCTTCAAA  432
             |||||||| |||||||||||||||||||||||||||||||||||||| |||||||
Sbjct  2078  GTTTATGATCTAAGTGACTTGCTGACCGATCCTTCTTGACCAAAGAAGCTTCAAA  2024
```
