# Supplementary material for: Occipital Horn Syndrome as a Result of Splice Site Mutations in ATP7A. No Activity of ATP7A Splice Variants Missing Exon 10 or Exon 15
Source: Front Mol Neurosci. 2021 Apr 21;14:532291. doi: 10.3389/fnmol.2021.532291 (PMC8097048; doi:10.3389/fnmol.2021.532291)
Supplement: Supplementary file 2 [file Data_Sheet_2.PDF]

MNK 411 P3

Sekvventeret: ex13 – ex14 – ex16 – ex17

Primer 13U:**ex13** – ex14 – **ex16** – ex17

```
Score =  279 bits (145),  Expect = 1e-71
 Identities = 160/165 (96%), Gaps = 1/165 (0%)
 Strand=Plus/Plus

Query  28    AACTTGTGGAC-AGGCACAAACATCACAGGCTCCTATCCGGCAGTTTGCAAACAAACTCA  86
             ||||||||||  |||||||||||||| |||||||||||| |||||||||| |||||||||
Sbjct  2898  AACTTGTGGAAGAGGCACAAACATCAAAGGCTCCTATCCAGCAGTTTGCAGACAAACTCA  2957

Query  87    GTGGCTATTTTGTTCCTTTTATTGTTTTTGTTTCCATTGCCACCCTCTTGGTATGGATTG  146
             ||||||||||||||||||||||||||||||||||||||||||||||||||||||||||||
Sbjct  2958  GTGGCTATTTTGTTCCTTTTATTGTTTTTGTTTCCATTGCCACCCTCTTGGTATGGATTG  3017

Query  147   TAATTGGATTTCTGAATTTTGAAATTGTGGAAACCTACTTTCCTG  191
             |||||||||||||||||||||||||||||||||||||||||||||
Sbjct  3018  TAATTGGATTTCTGAATTTTGAAATTGTGGAAACCTACTTTCCTG  3062

Score =  483 bits (251),  Expect = 4e-133
 Identities = 251/251 (100%), Gaps = 0/251 (0%)
 Strand=Plus/Plus

Query  191   GTAAAGGTAGTGGTATTTGATAAGACTGGAACCATTACTCACGGAACCCCAGTGGTGAAT  250
             ||||||||||||||||||||||||||||||||||||||||||||||||||||||||||||
Sbjct  3257  GTAAAGGTAGTGGTATTTGATAAGACTGGAACCATTACTCACGGAACCCCAGTGGTGAAT  3316

Query  251   CAAGTAAAGGTTCTAACTGAAAGTAACAGAATATCACACCATAAAATCTTGGCCATTGTG  310
             ||||||||||||||||||||||||||||||||||||||||||||||||||||||||||||
Sbjct  3317  CAAGTAAAGGTTCTAACTGAAAGTAACAGAATATCACACCATAAAATCTTGGCCATTGTG  3376

Query  311   GGAACTGCTGAAAGTAACAGTGAACACCCTCTAGGAACAGCCATAACCAAATATTGCAAA  370
             ||||||||||||||||||||||||||||||||||||||||||||||||||||||||||||
Sbjct  3377  GGAACTGCTGAAAGTAACAGTGAACACCCTCTAGGAACAGCCATAACCAAATATTGCAAA  3436

Query  371   CAGGAGCTGGACACTGAAACCTTGGGTACCTGCATAGATTTCCAGGTTGTGCCAGGCTGT  430
             ||||||||||||||||||||||||||||||||||||||||||||||||||||||||||||
Sbjct  3437  CAGGAGCTGGACACTGAAACCTTGGGTACCTGCATAGATTTCCAGGTTGTGCCAGGCTGT  3496

Query  431   GGTATTAGCTG  441
             |||||||||||
Sbjct  3497  GGTATTAGCTG  3507
```

Primer 13L:**ex17** - ex16 - **ex14** - ex13

```
Score =  352 bits (183),  Expect = 7e-94
 Identities = 185/186 (99%), Gaps = 0/186 (0%)
 Strand=Plus/Minus

Query  31    CTCCTGTTTGCAATATTTGGTTATGGCTGTTCCTAGAGGGTGTTCACTGTTACTTTCAAC  90
             |||||||||||||||||||||||||||||||||||||||||||||||||||||||||| |
Sbjct  3442  CTCCTGTTTGCAATATTTGGTTATGGCTGTTCCTAGAGGGTGTTCACTGTTACTTTCAGC  3383

Query  91    AGTTCCCACAATGGCCAAGATTTTATGGTGTGATATTCTGTTACTTTCAGTTAGAACCTT  150
             ||||||||||||||||||||||||||||||||||||||||||||||||||||||||||||
Sbjct  3382  AGTTCCCACAATGGCCAAGATTTTATGGTGTGATATTCTGTTACTTTCAGTTAGAACCTT  3323

Query  151   TACTTGATTCACCACTGGGGTTCCGTGAGTAATGGTTCCAGTCTTATCAAATACCACTAC  210
             ||||||||||||||||||||||||||||||||||||||||||||||||||||||||||||
Sbjct  3322  TACTTGATTCACCACTGGGGTTCCGTGAGTAATGGTTCCAGTCTTATCAAATACCACTAC  3263

Query  211   CTTTAC  216
             ||||||
Sbjct  3262  CTTTAC  3257


Score =  410 bits (213),  Expect = 3e-111
 Identities = 219/222 (98%), Gaps = 0/222 (0%)
 Strand=Plus/Minus

Query  216   CAGGAAAGTAGGTTTCCACAATTTCAAAATTCAGAAATCCAATTACAATCCATACCAAGA  275
             ||||||||||||||||||||||||||||||||||||||||||||||||||||||||||||
Sbjct  3062  CAGGAAAGTAGGTTTCCACAATTTCAAAATTCAGAAATCCAATTACAATCCATACCAAGA  3003

Query  276   GGGGGGCAATGGAAACAAAAACAATAAAAGGAACAAAATAGCCACTGAGTTTGTCTGCAA  335
             ||| ||||||||||||||||||||||||||||||||||||||||||||||||||||||||
Sbjct  3002  GGGTGGCAATGGAAACAAAAACAATAAAAGGAACAAAATAGCCACTGAGTTTGTCTGCAA  2943

Query  336   ACTGCTGGATAGGAGCCTTTGATGTTTGTGCCTCTTCCACAAGTTTGACAATTTGAGAAA  395
             ||||||||||||||||||||||||||||||||||||||||||||||||||||||||||||
Sbjct  2942  ACTGCTGGATAGGAGCCTTTGATGTTTGTGCCTCTTCCACAAGTTTGACAATTTGAGAAA  2883

Query  396   GGGTTGTGTCTGCTCCAACATATGTTGCGCAAATAAGCAGTG  437
             ||||||||||||||||||||| ||||||||| ||||||||||
Sbjct  2882  GGGTTGTGTCTGCTCCAACATGTGTTGCGCAGATAAGCAGTG  2841
```
