# Supplementary material for: Occipital Horn Syndrome as a Result of Splice Site Mutations in ATP7A. No Activity of ATP7A Splice Variants Missing Exon 10 or Exon 15
Source: Front Mol Neurosci. 2021 Apr 21;14:532291. doi: 10.3389/fnmol.2021.532291 (PMC8097048; doi:10.3389/fnmol.2021.532291)
Supplement: Supplementary file 3 [file Data_Sheet_3.PDF]

MNK 19

P4

Samlet sekventering: ex 8 - ex 9 - ex 11

Primer 8U: **ex8** - ex9 - **ex11**

```
Score =  462 bits (240),  Expect = 1e-126
 Identities = 258/262 (98%), Gaps = 2/262 (0%)
 Strand=Plus/Plus

Query  6     AGCAGGTCACTTAGATCATAA-CGAGAA-TAAGACAATGGAGACGGTCTTTTCTTGTGAG  63
             |||| |||||||||||||||| |||||| |||||||||||||||||||||||||||||||
Sbjct  2056  AGCAAGTCACTTAGATCATAAACGAGAAATAAGACAATGGAGACGGTCTTTTCTTGTGAG  2115

Query  64    TCTGTTTTTCTGTATTCCTGTAATGGGGCTGATGATATATATGATGGTTATGGACCACCA  123
             |||||||||||||||||||||||||||||||||| |||||||||||||||||||||||||
Sbjct  2116  TCTGTTTTTCTGTATTCCTGTAATGGGGCTGATGACATATATGATGGTTATGGACCACCA  2175

Query  124   CTTTGCAACTCTTCACCATAATCAAAACATGAGTAAAGAAGAAATGATCAACCTTCATTC  183
             ||||||||||||||||||||||||||||||||||||||||||||||||||||||||||||
Sbjct  2176  CTTTGCAACTCTTCACCATAATCAAAACATGAGTAAAGAAGAAATGATCAACCTTCATTC  2235

Query  184   TTCTATGTTCCTGGAGCGCCAGATTCTTCCAGGATTGTCTGTTATGAATTTGCTGTCCTT  243
             ||||||||||||||||||||||||||||||||||||||||||||||||||||||||||||
Sbjct  2236  TTCTATGTTCCTGGAGCGCCAGATTCTTCCAGGATTGTCTGTTATGAATTTGCTGTCCTT  2295

Query  244   TTTATTGTGTGTACCTGTACAG  265
             ||||||||||||||||||||||
Sbjct  2296  TTTATTGTGTGTACCTGTACAG  2317


Score =  302 bits (157),  Expect = 2e-78
 Identities = 157/157 (100%), Gaps = 0/157 (0%)
 Strand=Plus/Plus

Query  264   AGGGCAAAACATCAGAGGCTCTTGCAAAGTTAATTTCACTACAAGCTACAGAAGCAACTA  323
             ||||||||||||||||||||||||||||||||||||||||||||||||||||||||||||
Sbjct  2550  AGGGCAAAACATCAGAGGCTCTTGCAAAGTTAATTTCACTACAAGCTACAGAAGCAACTA  2609

Query  324   TTGTAACTCTTGATTCTGATAATATCCTCCTCAGTGAAGAACAAGTGGATGTGGAACTTG  383
             ||||||||||||||||||||||||||||||||||||||||||||||||||||||||||||
Sbjct  2610  TTGTAACTCTTGATTCTGATAATATCCTCCTCAGTGAAGAACAAGTGGATGTGGAACTTG  2669

Query  384   TACAACGTGGAGATATCATTAAAGTAGTTCCAGGAGG  420
             |||||||||||||||||||||||||||||||||||||
Sbjct  2670  TACAACGTGGAGATATCATTAAAGTAGTTCCAGGAGG  2706
```

Primer 12L: **ex12** – ex11 – **ex9** – ex 8

```
Score =  227 bits (118),  Expect = 4e-56
 Identities = 118/118 (100%), Gaps = 0/118 (0%)
 Strand=Plus/Minus

Query  18    AGTTCCACATCCACTTGTTCTTCACTGAGGAGGATATTATCAGAATCAAGAGTTACAATA  77
             ||||||||||||||||||||||||||||||||||||||||||||||||||||||||||||
Sbjct  2667  AGTTCCACATCCACTTGTTCTTCACTGAGGAGGATATTATCAGAATCAAGAGTTACAATA  2608

Query  78    GTTGCTTCTGTAGCTTGTAGTGAAATTAACTTTGCAAGAGCCTCTGATGTTTTGCCCT  135
             ||||||||||||||||||||||||||||||||||||||||||||||||||||||||||
Sbjct  2607  GTTGCTTCTGTAGCTTGTAGTGAAATTAACTTTGCAAGAGCCTCTGATGTTTTGCCCT  2550


Score =  554 bits (288),  Expect = 2e-154
 Identities = 292/294 (99%), Gaps = 0/294 (0%)
 Strand=Plus/Minus

Query  134   CTGTACAGGTACACACAATAAAAAGGACAGCAAATTCATAACAGACAATCCTGGAAGAAT  193
             ||||||||||||||||||||||||||||||||||||||||||||||||||||||||||||
Sbjct  2317  CTGTACAGGTACACACAATAAAAAGGACAGCAAATTCATAACAGACAATCCTGGAAGAAT  2258

Query  194   CTGGCGCTCCAGGAACATAGAAGAATGAAGGTTGATCATTTCTTCTTTACTCATGTTTTG  253
             ||||||||||||||||||||||||||||||||||||||||||||||||||||||||||||
Sbjct  2257  CTGGCGCTCCAGGAACATAGAAGAATGAAGGTTGATCATTTCTTCTTTACTCATGTTTTG  2198

Query  254   ATTATGGTGAAGAGTTGCAAAGTGGTGGTCCATAACCATCATATATATCATCAGCCCCAT  313
             ||||||||||||||||||||||||||||||||||||||||||||||| ||||||||||||
Sbjct  2197  ATTATGGTGAAGAGTTGCAAAGTGGTGGTCCATAACCATCATATATGTCATCAGCCCCAT  2138

Query  314   TACAGGAATACAGAAAAACAGACTCACAAGAAAAGACCGTCTCCATTGTCTTATTTCTCG  373
             ||||||||||||||||||||||||||||||||||||||||||||||||||||||||||||
Sbjct  2137  TACAGGAATACAGAAAAACAGACTCACAAGAAAAGACCGTCTCCATTGTCTTATTTCTCG  2078

Query  374   TTTATGATCTAAGTGACTTGCTGACCGATCCTTCTTGACCAAAGAAGTTTCAAA  427
             ||||||||||||||||||||||||||||||||||||||||||||||| ||||||
Sbjct  2077  TTTATGATCTAAGTGACTTGCTGACCGATCCTTCTTGACCAAAGAAGCTTCAAA  2024
```
