# Supplementary material for: Occipital Horn Syndrome as a Result of Splice Site Mutations in ATP7A. No Activity of ATP7A Splice Variants Missing Exon 10 or Exon 15
Source: Front Mol Neurosci. 2021 Apr 21;14:532291. doi: 10.3389/fnmol.2021.532291 (PMC8097048; doi:10.3389/fnmol.2021.532291)
Supplement: Supplementary file 4 [file Data_Sheet_4.PDF]

MNK 409 P2

Sekventeret:ex13 -ex 14 - ex16 - ex17

Primer 13U: **ex13** -ex 14 - **ex16** - ex17

```
Score =  308 bits (160),  Expect = 2e-80
 Identities = 162/163 (99%), Gaps = 0/163 (0%)
 Strand=Plus/Plus

Query  32    CTTGTGGAAGAGGCACAAACATCAAAGGCTCCTATCCGGCAGTTTGCAGACAAACTCAGT  91
             ||||||||||||||||||||||||||||||||||||| ||||||||||||||||||||||
Sbjct  2900  CTTGTGGAAGAGGCACAAACATCAAAGGCTCCTATCCAGCAGTTTGCAGACAAACTCAGT  2959

Query  92    GGCTATTTTGTTCCTTTTATTGTTTTTGTTTCCATTGCCACCCTCTTGGTATGGATTGTA  151
             ||||||||||||||||||||||||||||||||||||||||||||||||||||||||||||
Sbjct  2960  GGCTATTTTGTTCCTTTTATTGTTTTTGTTTCCATTGCCACCCTCTTGGTATGGATTGTA  3019

Query  152   ATTGGATTTCTGAATTTTGAAATTGTGGAAACCTACTTTCCTG  194
             |||||||||||||||||||||||||||||||||||||||||||
Sbjct  3020  ATTGGATTTCTGAATTTTGAAATTGTGGAAACCTACTTTCCTG  3062


Score =  469 bits (244),  Expect = 5e-129
 Identities = 244/244 (100%), Gaps = 0/244 (0%)
 Strand=Plus/Plus

Query  194   GTAAAGGTAGTGGTATTTGATAAGACTGGAACCATTACTCACGGAACCCCAGTGGTGAAT  253
             ||||||||||||||||||||||||||||||||||||||||||||||||||||||||||||
Sbjct  3257  GTAAAGGTAGTGGTATTTGATAAGACTGGAACCATTACTCACGGAACCCCAGTGGTGAAT  3316

Query  254   CAAGTAAAGGTTCTAACTGAAAGTAACAGAATATCACACCATAAAATCTTGGCCATTGTG  313
             ||||||||||||||||||||||||||||||||||||||||||||||||||||||||||||
Sbjct  3317  CAAGTAAAGGTTCTAACTGAAAGTAACAGAATATCACACCATAAAATCTTGGCCATTGTG  3376

Query  314   GGAACTGCTGAAAGTAACAGTGAACACCCTCTAGGAACAGCCATAACCAAATATTGCAAA  373
             ||||||||||||||||||||||||||||||||||||||||||||||||||||||||||||
Sbjct  3377  GGAACTGCTGAAAGTAACAGTGAACACCCTCTAGGAACAGCCATAACCAAATATTGCAAA  3436

Query  374   CAGGAGCTGGACACTGAAACCTTGGGTACCTGCATAGATTTCCAGGTTGTGCCAGGCTGT  433
             ||||||||||||||||||||||||||||||||||||||||||||||||||||||||||||
Sbjct  3437  CAGGAGCTGGACACTGAAACCTTGGGTACCTGCATAGATTTCCAGGTTGTGCCAGGCTGT  3496

Query  434   GGTA  437
             ||||
Sbjct  3497  GGTA  3500
```

Primer 17L: **ex17** – ex16 – **ex14** – ex13

```
Score =  367 bits (191),  Expect = 2e-98
 Identities = 198/199 (99%), Gaps = 1/199 (0%)
 Strand=Plus/Minus

Query  18    TTTCAGTGTC-AGCTCCTGTTTGCAATATTTGGTTATGGCTGTTCCTAGAGGGTGTTCAC  76
             |||||||||| |||||||||||||||||||||||||||||||||||||||||||||||||
Sbjct  3455  TTTCAGTGTCCAGCTCCTGTTTGCAATATTTGGTTATGGCTGTTCCTAGAGGGTGTTCAC  3396

Query  77    TGTTACTTTCAGCAGTTCCCACAATGGCCAAGATTTTATGGTGTGATATTCTGTTACTTT  136
             ||||||||||||||||||||||||||||||||||||||||||||||||||||||||||||
Sbjct  3395  TGTTACTTTCAGCAGTTCCCACAATGGCCAAGATTTTATGGTGTGATATTCTGTTACTTT  3336

Query  137   CAGTTAGAACCTTTACTTGATTCACCACTGGGGTTCCGTGAGTAATGGTTCCAGTCTTAT  196
             ||||||||||||||||||||||||||||||||||||||||||||||||||||||||||||
Sbjct  3335  CAGTTAGAACCTTTACTTGATTCACCACTGGGGTTCCGTGAGTAATGGTTCCAGTCTTAT  3276

Query  197   CAAATACCACTACCTTTAC  215
             |||||||||||||||||||
Sbjct  3275  CAAATACCACTACCTTTAC  3257

Score =  433 bits (225),  Expect = 4e-118
 Identities = 227/228 (99%), Gaps = 0/228 (0%)
 Strand=Plus/Minus

Query  215   CAGGAAAGTAGGTTTCCACAATTTCAAAATTCAGAAATCCAATTACAATCCATACCAAGA  274
             ||||||||||||||||||||||||||||||||||||||||||||||||||||||||||||
Sbjct  3062  CAGGAAAGTAGGTTTCCACAATTTCAAAATTCAGAAATCCAATTACAATCCATACCAAGA  3003

Query  275   GGGTGGCAATGGAAACAAAAACAATAAAAGGAACAAAATAGCCACTGAGTTTGTCTGCAA  334
             ||||||||||||||||||||||||||||||||||||||||||||||||||||||||||||
Sbjct  3002  GGGTGGCAATGGAAACAAAAACAATAAAAGGAACAAAATAGCCACTGAGTTTGTCTGCAA  2943

Query  335   ACTGCTGGATAGGAGCCTTTGATGTTTGTGCCTCTTCCACAAGTTTGACAATTTGAGAAA  394
             ||||||||||||||||||||||||||||||||||||||||||||||||||||||||||||
Sbjct  2942  ACTGCTGGATAGGAGCCTTTGATGTTTGTGCCTCTTCCACAAGTTTGACAATTTGAGAAA  2883

Query  395   GGGTTGTGTCTGCTCCAACATGTGTTGCGCAAATAAGCAGTGACCCGT  442
             |||||||||||||||||||||||||||||||| |||||||||||||||
Sbjct  2882  GGGTTGTGTCTGCTCCAACATGTGTTGCGCAGATAAGCAGTGACCCGT  2835
```
